# Crystal structure of human Fanconi-associated nuclease 1

**DOI:** 10.1007/s13238-014-0128-y

**Published:** 2014-12-31

**Authors:** Peng-xian Yan, Yan-gao Huo, Tao Jiang

**Affiliations:** 1 National Laboratory of Biomacromolecules, Institute of Biophysics, Chinese Academy of Sciences, Beijing, 100101 China; 2 University of Chinese Academy of Sciences, Beijing, 100039 China

**Dear Editor,**

DNA interstrand crosslinks (ICLs) are toxic to cells because they covalently link the two strands in the duplex. ICLs can be induced by crosslinking agents and by cell endogenous metabolites, and physically prevent transcription and replication in both directions (MacKay et al., 2010). This type of DNA damage must be properly repaired to maintain genomic and cellular integrity, and this repair is primarily accomplished by the Fanconi anemia pathway (FA pathway) (Moldovan and D’Andrea, 2009). FA is an inherited recessive developmental and cancer predisposition syndrome that can be caused by defects in any of 16 FANC proteins (Chaudhury et al., 2014). The removal of ICLs depends on numerous components involved in multiple DNA repair pathways such as nucleotide excision repair (NER), mismatch repair (MMR), translesion synthesis (TLS) and homologous recombination (HR), and several models have been previously proposed (Fu et al., 2011; Huang et al., 2013; Kratz et al., 2010). Among these models, flap-shaped DNA or splayed duplex DNA structures are expected to exist during the multiple steps of repair. Consequently, a number of nucleases have been suggested to function in ICL repair, including MUS81-EME1, XPF-ERCC1 and SLX4-SLX1 (Douwel et al., 2014).

In 2010, four groups identified a novel FA-associated nuclease, FAN1, that directly binds to monoubiquitinated FANCD_2_, participating in ICL repair (Kratz et al., 2010; Liu et al., 2010; MacKay et al., 2010; Smogorzewska et al., 2010). FAN1 is a structure-specific nuclease and possesses preferential endonuclease activity toward 5′ flap structures and a weaker 5′-3′ exonuclease activity *in vitro* and has been hypothesized to cleave DNA in multiple steps (MacKay et al., 2010; O’Donnell and Durocher, 2010). The FAN1 nuclease is recruited to the damage foci through its UBZ domain and acts as an effector by executing one or more DNA strand incisions during ICL repair (Zhang and Walter, 2014). Previous studies have indicated that the depletion of FAN1 or mutations that defect the nuclease activity cause hypersensitivity to ICL-inducing agents and defects in HR (MacKay et al., 2010), suggesting its significance for efficient ICL repair. Moreover, subsequent studies have demonstrated that mutations in FAN1 cause karyomegalic interstitial nephritis (KIN), implicating a vital role for FAN1 in kidney function and a correlation between DNA damage and chronic kidney failure (Lans and Hoeijmakers, 2012; Zhou et al., 2012).

Here, we report the crystal structure of human FAN1 lacking the highly flexible UBZ domain (referred to hereafter as hFAN1 for brevity). The crystal structure of hFAN1 was determined at 2.8 Å resolution using the single-wavelength anomalous diffraction (SAD) method. The asymmetric unit contains one molecule encompassing residues 371–1010 with four disordered loops (residues 506–516, 557–569, 755–765 and 792–809). Data collection and refinement statistics are summarized in Table 1 in supplementary materials.

The overall structure of hFAN1 consists of an SAP-containing N-terminal domain (NTD, residues 371–594), a middle tetratricopeptide repeat domain (TPR domain, residues 595–772) and a C-terminal viral replication and repair nuclease domain (VRR_nuc domain, residues 773–1010) and folds into the shape of a short handled scoop (Fig. 1A and 1B). The NTD domain can be divided into two regions: an SAP subdomain (α7–10) and a compact structure consisting of an N-terminal 6-helix-bundle together with a wing formed by α11 and the loop between α11 and α12, termed the “wedge” in *Pseudomonas aeruginosa* FAN1 (PaFAN1) (Gwon et al., 2014). The TPR domain consists of nine α-helices (α12–20) that form a solenoid domain as a bridge between the NTD and VRR_nuc domains. The VRR_nuc catalytic domain lying at the C-terminus contains a typic VRR_nuc domain with a helical insertion consisting of 6 α-helices (α22–27) inserted between β4 and β5.

**Figure 1:**
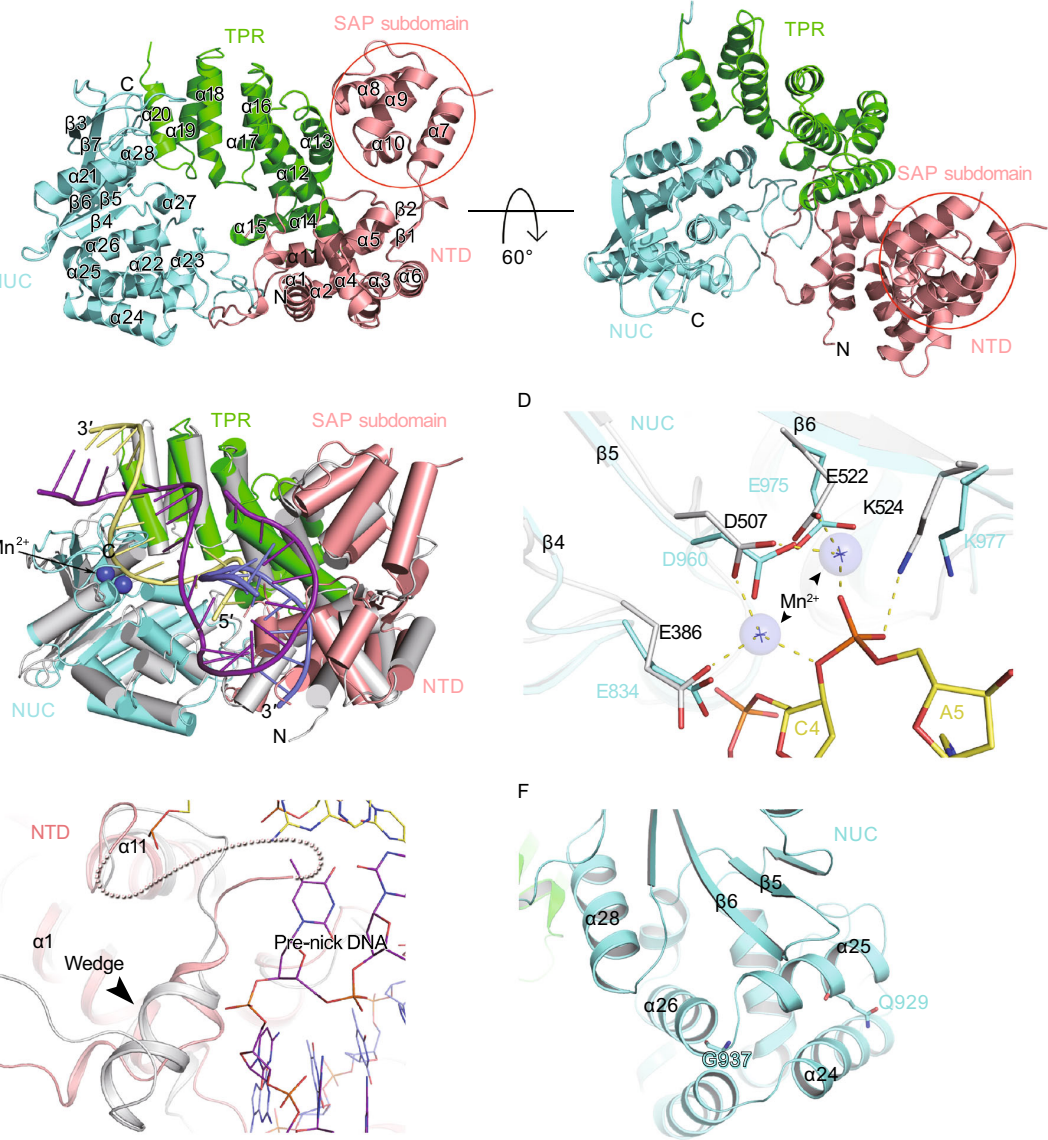
**Overall structure of hFAN1 and structure comparison of hFAN1 with PaFAN1**. (A) Overall structure of hFAN1. The SAP-containing NTD is colored salmon, the TPR domain is colored green, and the VRR_nuc domain is colored cyan. (B) Alternative view of (A) rotated 60°. (C) Structural comparison of hFAN1 and PaFAN1-DNA-Mn^2+^. PaFAN1 is colored gray with Mn^2+^ ions shown as blue spheres. (D) The active sites of hFAN1 and Mn^2+^-bound PaFAN1. The key residues contributing to the nuclease activity are labeled and shown as sticks. (E) Superposition of hFAN1 and the pre-nick DNA-binding “wedge” illustrated in PaFAN1. The loop of hFAN1 between α11 and the counterpart of “wedge” in PaFAN1 is disordered and represented by dotted lines. (F) The location of germline mutations (Q929 and G937) related to KIN in VRR_nuc insertion of hFAN1

To identify the conserved and divergent regions in hFAN1 and PaFAN1 (Gwon et al., 2014), we superpose hFAN1 with PaFAN1 in complex with 5′ flap DNA. Structural comparison indicates that despite slight local secondary structure changes, the overall structures are highly similar (Fig. 1C). In the active site of PaFAN1, residues D507, E522 and K524 of the typical PDX_n_(D/E)XK motif, together with a nearby residue, E386, coordinate the dimetal group, and these four residues are strictly conserved in hFAN1 (D960, E975, K977 and E834) (Fig. 1D). In addition, the nearly identical orientation of these residues suggests that they may share a common DNA cleavage mechanism.

Moreover, the overall DNA binding pattern of hFAN1 appears to share high similarity with PaFAN1, which is exemplified by the observation that 12 of 21 basic DNA-binding residues of PaFAN1 along the DNA elongation path are strictly conserved in hFAN1. These residues are scattered in all three domains of hFAN1, namely Y374, R420, R424, K425 and K493 in the N-terminal domain; R668, R710 and R752 in the TPR domain; and R955, G957, K977 and R982 in the VRR_nuc domain. These similarities allow us to construct a binding model of 5′ flap DNA with hFAN1 characterized by extensive interactions with all three domains in a sharply bent DNA conformation.

In addition, the notably disordered loop between α11 and α12 is close to the equivalent position of the “wedge” that protrudes into the substrate junction in the PaFAN1 structure (Gwon et al., 2014), suggesting that this loop in hFAN1 may be involved in DNA-induced conformational changes (Fig. 1E).

Although the overall fold and binding mode for 5′ flap DNA of PaFAN1 and hFAN1 are similar, some major differences exist (Fig. 2A).

**Figure 2:**
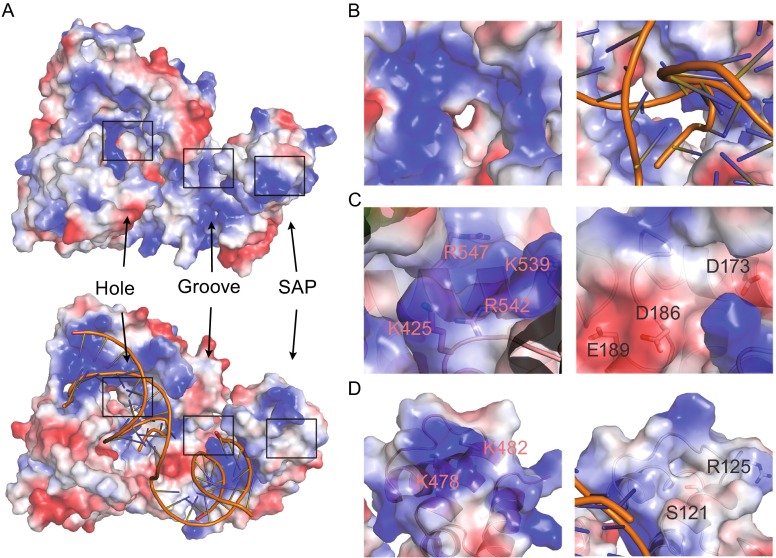
**Comparison of the electrostatic surface potential of hFAN1 and PaFAN1**. (A) The major differences between hFAN1 (upper) and PaFAN1 (lower) are highlighted with squares. Detailed views of the hole, groove and SAP subdomain are shown in (B), (C) and (D), respectively, with hFAN1 shown in the left panels and PaFAN1 in the right panels

First, in the vicinity of the 5′ flap binding site, PaFAN1 possesses an elliptical hole with a size that is sufficient to allow only the passage of ssDNA, while the flap DNA end does not thread into this hole but turns back. In contrast, at the equivalent position in hFAN1, the hole is much smaller and unlikely to allow ssDNA passage (Fig. 2B). Thus, our structure supports the notion that hFAN1 is unlikely to sequester 5′ flap DNA via a threading mechanism.

Second, significant differences in the electrostatic surface potential for PaFAN1 and hFAN1 are found in the groove between the N-terminal domain and the TPR domain. The electrostatic surface potential is highly negative in PaFAN1, which arises from the clustering of negatively charged residues including D173, D186 and E189, whereas the electrostatic surface potential of hFAN1 is positive, with contributions from residues K425, K539, R542 and R547 (Fig. 2C). Moreover, the SAP subdomains of PaFAN1 and hFAN1 that flank the groove also exhibit notable differences in the electrostatic surface potential (Fig. 2D), with different types of residues and side chain orientations (K478 and K482 in hFAN1 and S121 and R125 in PaFAN1).

Previous studies have indicated that the substrate specificities of PaFAN1 and hFAN1 are largely similar, whereas some minor differences in the substrate specificities were observed (Gwon et al., 2014). For instance, PaFAN1 exhibits weaker activity toward a splayed-arm substrate than hFAN1. We speculate that the noted structural differences may be related to these minor differences in substrate specificity, which warrants future studies.

The structure reported herein provides insights into key structural elements of hFAN1 and sheds new light on the molecular basis of related diseases. For example, Zhou et al. identified that three missense mutations, Q929P, G937D and D960Q in hFAN1 can cause KIN, a kidney disease with renal tabular degeneration (Zhou et al., 2012). Mapping of these residues onto our structure indicates that only D960 is involved in DNA binding and incision; the remaining two residues, Q929 and G937, are located at the VRR_nuc insertion region, which is proposed to prevent HJ resolvase-like dimerization (Fig. 1F). Thus, it is tempting to speculate that mutations of these two residues may affect the local mobility or protein-protein interactions; however, future studies are necessary to confirm this speculation.

The coordinates and the structure factors of human FAN1 have been deposited in the Protein Data Bank under the accession code 4RY3.

## Footnotes

We thank the staff at the BL17U beamline of the SSRF (Shanghai Synchrotron Radiation Facility) in China and the BL-17A beamline of the Photon Factory in Japan for technical assistance during data collection. This work was supported by grants from the National Basic Research Program (973 Program) (No. 2011CB910302), the Strategic Priority Research Program (XDB08010301), and the National Natural Science Foundation of China (Grant No. 31025009).

During the preparation of this manuscript, an article on the crystal structure of almost the same region of hFAN1 was published online (Wang et al., 2014).

Peng-xian Yan, Yan-gao Huo and Tao Jiang declare that they have no conflict of interest. This article does not contain any studies with human or animal subjects performed by any of the authors.

## Electronic supplementary material

Below is the link to the electronic supplementary material.

Supplementary material 1 (DOCX 33 kb)
