# Supplementary material for: Crystal structure of human Fanconi-associated nuclease 1
Source: Protein Cell. 2014 Dec 31;6(3):225–8. doi: 10.1007/s13238-014-0128-y (PMC4348246; doi:10.1007/s13238-014-0128-y)
Supplement: Supplementary file 1 — Supplementary material 1 (DOCX 33 kb) [file 13238_2014_128_MOESM1_ESM.docx]

**Crystal structure of human Fanconi-associated nuclease 1**

Peng-xian Yan^1, 2^, Yan-gao Huo^1^*, Tao Jiang^1^*

^1^National Laboratory of Biomacromolecules, Institute of Biophysics, Chinese Academy of Sciences, Beijing 100101, China

^2^University of Chinese Academy of Sciences, 19A Yuquan Road, Shijingshan District, Beijing 100039, China

Materials and methods

*Protein expression and purification*

Human FAN1 (residues 371-1010) was cloned into the pPICZ B vector between the *Eco*RI and *Kpn*I restriction sites with a GFP fluorescent label followed by a 6-His tag at the C-terminus. The plasmid was linearized with *Pme*I and transformed into the X33 strain of *Pichia pastoris* for expression. The expressed protein was firstly purified using nickel affinity chromatography, and the GFP and His tag were then removed via TEV proteolysis. Afterwards the protein was purified to homogeneity by size exclusion chromatography using Sepharose S and Superdex 200 10/300 columns and concentrated to a final concentration of 6 mg/mL in 50 mM Tris-HCl, pH 7.5, 1 M KCl, and 5 mM dithiothreitol for crystallization.

*Crystallization and data collection*

Crystals were grown at 4°C using the hanging drop vapor diffusion method by mixing 1 μL protein solution with 1 μL reservoir solution containing 2.5 M sodium acetate, 0.1 M Bis-Tris propane, pH 8.2, and 10% glycerol. The heavy-atom derivative crystals were obtained by adding potassium tetracyanoplatinate hydrate to the hanging drop to a final concentration of 2 mM, and the crystals were soaked for approximately 24 h. The crystals were flash frozen in liquid nitrogen for data collection. Diffraction data were collected at beamline 17U (BL17U) at the Shanghai Synchrotron Radiation Facility (SSRF) and processed using HKL2000 (Otwinowski and Minor, 1997).

*Structure determination and refinement*

The crystal belonged to the space group *P*2_1_3 with one monomer in the asymmetric unit. The initial phases of the heavy atom were obtained with the HKL2MAP program (Pape and Schneider, 2004; Schwarzenbach et al., 2010) using the platinum-derivatized crystals. Electron density maps were calculated using the single-wavelength anomalous dispersion method (SAD) by PHENIX (Adams et al., 2010; Terwilliger et al., 2009). Model building was performed using COOT (Emsley et al., 2010), and refinement was performed using REFMAC5 (Murshudov et al., 1997). The final model was refined to 2.8 Å with *R_work_* and *R_free_* values of 20.8% and 24.3%, respectively. Structural figures were rendered using PyMol (<http://www.pymol.org>).

TableS1. Data collection, structure determination, and refinement statistics.

| hFAN1 (Pt-derivative) | |
| --- | --- |
| Data collection | |
| Space group | P2_1_3 |
| Wavelength (Å) | 1.068 |
| Resolution (Å) | 20-2.8 |
| Cell dimensions  *a, b, c* (Å)  *α, β, γ* (°) | 161.1, 161.1, 161.1  90, 90, 90 |
| Unique reflections | 34264 (3317) |
| I/σ(I) | 23.8 (3.6) |
| Completeness (%) | 99.61 (97.02) |
| Redundancy | 10.1 (9.8) |
| *R_merge_* (%)*^a^* | 5.8 (68) |
| Wilson B-factor (Å) | 34.15 |
| Refinement | |
| *R*_work_(%)*^b^*  *R*_free_(%)*^c^* | 20.8  24.3 |
| Average B factor (Å^2^) | 49.0 |
| Root mean square deviations  Bond lengths (Å)  Bond angles (°) | 0.011  1.33 |
| Ramachandran plot  Most favored (%)  Additionally allowed (%)  Generously allowed (%)  Disallowed | 90.2  9.8  0  0 |

Numbers in parentheses represent statistics for the highest resolution shell.

*^a^ R*_merge_ = Σ| *I - < I >*| / Σ*I* , where *I* is the measured intensity for reflections with indices *hkl*.

*^b^ R*_work_ = Σ|| F_obs_| - |F_calc_|| / Σ|F*_obs_*|.

*^c^* *R*_free_= R factor for a selected subset (5%) of the reflections that were not included in prior refinement calculations.

Reference

Adams, P.D., Afonine, P.V., Bunkoczi, G., Chen, V.B., Davis, I.W., Echols, N., Headd, J.J., Hung, L.W., Kapral, G.J., Grosse-Kunstleve, R.W.*, et al.* (2010). PHENIX: a comprehensive Python-based system for macromolecular structure solution. Acta Crystallogr D *66*, 213-221.

Emsley, P., Lohkamp, B., Scott, W.G., and Cowtan, K. (2010). Features and development of Coot. Acta Crystallogr D *66*, 486-501.

Murshudov, G.N., Vagin, A.A., and Dodson, E.J. (1997). Refinement of macromolecular structures by the maximum-likelihood method. Acta Crystallogr D *53*, 240-255.

Otwinowski, Z., and Minor, W. (1997). Processing of X-ray diffraction data collected in oscillation mode. Method Enzymol *276*, 307-326.

Pape, T., and Schneider, T.R. (2004). HKL2MAP: a graphical user interface for macromolecular phasing with SHELX programs. J Appl Crystallogr *37*, 843-844.

Schwarzenbach, D., Kostorz, G., McMahon, B., and Strickland, P. (2010). SHELX makes an impact. Acta Crystallogr A *66*, 631-631.

Terwilliger, T.C., Adams, P.D., Read, R.J., Mccoy, A.J., Moriarty, N.W., Grosse-Kunstleve, R.W., Afonine, P.V., Zwart, P.H., and Hung, L.W. (2009). Decision-making in structure solution using Bayesian estimates of map quality: the PHENIX AutoSol wizard. Acta Crystallogr D *65*, 582-601.
